# Carnosine‐Related Metabolism in Rat Cardiomyocytes and Human Heart Tissue

**DOI:** 10.1096/fj.202504676R

**Published:** 2026-07-15

**Authors:** Jade V. Creighton, Lívia de Souza Gonçalves, Saulo Gil, Bianca Scigliano Vargas, Leonardo Jensen, Marisa Helena Gennari de Medeiros, Hamilton Roschel, Mark D. Turner, Craig L. Doig, Guilherme Giannini Artioli, Craig Sale

**Affiliations:** ^1^ Centre for Systems Health and Integrated Metabolic Research (SHiMR), School of Science and Technology Nottingham Trent University Nottingham UK; ^2^ Department of Pediatrics, Division of Pediatric University of California San Francisco California USA; ^3^ Applied Physiology & Nutrition Research Group, School of Physical Education and Sport, Faculdade de Medicina, Divisão de Reumatologia Universidade de São Paulo São Paulo Brazil; ^4^ Departamento de Bioquímica, Instituto de Química Universidade de São Paulo São Paulo Brazil; ^5^ Laboratorio de Hipertensao do Instituto do Coraçao do Hospital das Clínicas da Faculdade de Medicina da Universidade São Paulo São Paulo Brazil; ^6^ Department of Anatomy, Institute of Biomedical Sciences University of São Paulo São Paulo Brazil; ^7^ Department of Sport and Exercise Sciences Manchester Metropolitan University Institute of Sport Manchester UK

**Keywords:** beta‐alanine, histidine‐containing dipeptides, human, myocardium, taurine

## Abstract

The therapeutic potential of carnosine in healthy and diseased heart models is promising; however, the understanding of carnosine and β‐alanine metabolism in cardiac tissue is lacking. Exploring how these compounds are metabolized in cardiac tissue is critical to determine their viability for in vivo supplementation studies. Two independent studies were conducted. Study 1 investigated the uptake of exogenous carnosine and β‐alanine in cardiomyocytes and the influence of this on the expression of carnosine‐ and β‐alanine‐related enzyme and transporter genes. Study 2 investigated whether human cardiac tissue expresses carnosine and β‐alanine metabolism proteins and the endogenous concentrations of carnosine and β‐alanine. In Study 1, differentiated H9c2 cells were treated with 0.1–10.0 mM of carnosine or β‐alanine for 4, 24 and 72 h. Gene expression was measured at 4 h using real‐time quantitative polymerase chain reaction, and amino acid and histidine‐containing dipeptides (HCD) concentrations were analyzed at all‐time points using an amino acid analyzer. In Study 2, post‐mortem human heart ventricle samples (*n* = 16) were analyzed for carnosine and β‐alanine using HPLC‐ESI^+^‐MS/MS, and metabolism‐related proteins using western blots. H9c2 cardiomyocytes expressed genes related to carnosine and β‐alanine metabolism, except for the β‐alanine transaminase, *AGXT2*. Carnosine supplementation did not affect gene expression, whereas β‐alanine decreased transporter *TAUT* and *PHT1* expression. Both exogenously supplied carnosine and β‐alanine were taken up and accumulated in cardiomyocytes. Carnosine‐ and β‐alanine‐related enzymes and transporters are present in human heart ventricles. In conclusion, cardiomyocytes and the human heart express enzymes and transporters required to uptake β‐alanine and synthesize carnosine.

## Graphical abstract

Carnosine‐related metabolism in rat cardiomyocytes and human heart tissue. Jade V Creighton, Lívia de Souza Gonçalves, Saulo Gil, Bianca Scigliano Vargas, Leonardo Jensen, Marisa Helena Gennari de Medeiros, Hamilton Roschel, Mark D Turner, Craig L Doig, Guilherme Giannini Artioli, Craig Sale. This translational paper investigated the uptake and metabolism of carnosine and β‐alanine in rat cardiomyocytes (H9c2 cells) and post‐mortem human cardiac samples. Both models expressed the enzymes and transporters required for β‐alanine uptake and carnosine synthesis. Exogenous carnosine and β‐alanine were taken up and accumulated in H9c2 cells in their intact forms; however, carnosine synthesis did not occur with exogenous β‐alanine. The graphical abstract was created with NIAID NIH BioArt Source and CorelDraw Home & Student X8.

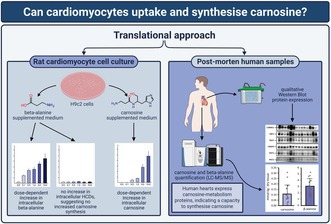

**Abbreviations**
Ca^2+^
: Calcium
Ct: Cycle threshold
DMEM: Dulbecco's modified eagle medium
ESI: Electrospray ionization
FBS: Foetal bovine serum
HCD: Histidine‐containing dipeptide
RT‐qPCR: Real‐time quantitative polymerase chain reaction
SRM: Selected reaction monitoring
ΔCt: Delta‐cycle threshold

## 1 Introduction

Carnosine (β‐alanyl‐L‐histidine) is a multifunctional histidine‐containing dipeptide (HCD), along with homocarnosine and its methylated (anserine and balenine) and acetylated (N‐acetylcarnosine) analogues [1, 2, 3]. HCDs are expressed in striated muscles, the brain, and the kidneys of several species of mammals, including humans and avians in a wide range of concentrations (~0.1–60 mM) [1, 4]. Since the isolation and characterization of carnosine in the early 1900s [5], our understanding of its metabolism and biological functions has developed significantly [1, 2, 3, 6]. Carnosine is the most abundant HCD in human skeletal muscle, with typical values of ~20–30 mmol٠kg^−1^ dry muscle [7, 8, 9, 10], whereas the concentrations of anserine, balenine, and N‐acetylcarnosine are at least 100 times lower [3, 11].

The major pathways of carnosine metabolism include its synthesis by carnosine synthase [12] and hydrolysis by carnosinases [13], alongside the activities of β‐alanine and peptide transporters [8, 14] and transaminases [14]. Although carnosine and other HCDs can be synthesized endogenously, the low availability of the precursor amino acid β‐alanine is the rate‐limiting factor for carnosine formation in skeletal muscle [2]. β‐alanine can be obtained from the diet, primarily through meat consumption [2]. Whilst muscle carnosine content in omnivores is approximately double that in vegetarians [15], β‐alanine supplementation (6.4 g/day; 4 weeks) provides 5–10 times more β‐alanine than a typical omnivorous diet, resulting in further increases of ~80%–150% in intramuscular carnosine [8, 9].

A well‐established property of carnosine in skeletal muscle is its role in acid–base regulation, which has been linked to increased exercise tolerance [16, 17]. Other functions with potential health applications have been explored in skeletal muscle and other tissues, including cardiac muscle, kidney and brain. These include the detoxification of reactive aldehydes [18], neutralization of reactive species [19], protection of cardiac muscle against oxidative stress [20, 21] and glycation‐end products [22], increased sensitivity of muscle contractile apparatus to calcium (Ca^2+^) [23], and improved Ca^2+^ release from the sarcoplasmic reticulum and Ca^2+^ dynamics [24, 25]. In cardiomyocytes, Zaloga et al. [26] were the first to show that carnosine can modulate excitation‐contraction coupling and improve contractility in isolated rat hearts. They demonstrated that carnosine increases free sarcoplasmic Ca^2+^ and alters ryanodin receptor 2 activity in a dose‐dependent manner (~1–10 mM). More recent studies using genetically modified rodent models to endogenously affect carnosine concentrations, conducted by our group [27] and others [28], provide further evidence that HCDs are key for normal cardiac function and exhibit protective effects under conditions of increased stress. These findings highlight carnosine's potential as a therapeutic target for cardiovascular diseases. Carnosine synthase knockout rats (*CARNS1* ^−/−^) displayed cardiac dysfunction due to Ca^2+^ handling and excitation‐contraction coupling disruption [27]. Zhao et al. [28] showed that cardiospecific overexpression of *CARNS1* protected the heart against the accumulation of toxic aldehydes, low pH, and energy imbalance after coronary ischemia–reperfusion.

Despite the key functions exerted by carnosine in the heart, its metabolism in cardiac cells is not fully understood, particularly regarding the transport of precursor amino acids to synthesize and metabolize HCDs and the synthesis of HCDs in response to increased availability of β‐alanine. Furthermore, it remains unknown whether human cardiac tissue expresses these molecules and whether cardiomyocytes can efficiently increase carnosine synthesis in response to increased availability of β‐alanine, with only one study having shown an increase in myocardial carnosine levels following oral β‐alanine administration in mice [28]. Establishing these fundamentals is essential for evaluating the potential therapeutic applications of carnosine. Therefore, we aimed to determine whether exogenous sources of β‐alanine and carnosine can be taken directly up by cardiomyocytes (Study 1) and to investigate whether key carnosine‐related enzymes and transporters are expressed in human heart (Study 2).

## 2 Material and Methods

### 2.1 Overview of the Experimental Approach

This paper comprises experiments from two complementary studies involving rat ventricular cardiomyoblast‐derived H9c2 cells (conducted at Nottingham Trent University, United Kingdom) and p*ostmortem* human heart samples (carried out at the University of São Paulo, Brazil). The first study sought to confirm the expression of carnosine and β‐alanine metabolism genes in H9c2 cardiomyocytes. H9c2 cells were then exposed to increasing concentrations of exogenous carnosine and β‐alanine for 4 to 72 h, with gene expression and amino acid analyses being performed to determine the mechanisms by which carnosine and β‐alanine availability influence carnosine homeostasis. Postmortem human heart samples were analyzed using HPLC‐MS/MS to identify the presence of β‐alanine and carnosine and using Western Blot to determine the expression of proteins associated with carnosine metabolism.

### 2.2 H9c2 Cardiomyocytes

#### 2.2.1 Cell Culture

Rat ventricular cardiomyoblast‐derived H9c2 cells (European Collection of Animal Cell Cultures, Porton Down, Salisbury, United Kingdom; kindly donated by Dr. John Dickenson, Nottingham Trent University) were cultured and maintained in T‐75 tissue culture flasks in growth media composed of Dulbecco's modified Eagle's medium (DMEM; Lonza, UK) supplemented with 10% foetal bovine serum (FBS) (Gibco, Thermo Fisher Scientific, UK), 2 mM L‐glutamine (Thermo Fisher Scientific, UK), and 1% (v/v) penicillin–streptomycin (Thermo Fisher Scientific, UK). Cells were incubated under standard conditions in a humidified incubator at 37°C and 5% CO_2_. Six biological replicates were completed for each experiment (see Supporting Information S1).

#### 2.2.2 Amino Acid and HCD Analysis

Amino acid concentrations (taurine, β‐alanine, and L‐histidine) and HCD concentrations (carnosine and anserine) in both cell lysates (intracellular) and culture media (extracellular) were determined using a Biochrom 30 high‐performance liquid chromatography ion exchange system (Biochrom, UK) with fluoraldehyde o‐phthaldialdehyde (OPA) post‐derivatisation. The protocol was adapted from Santos et al. [29].

H9c2 cells were seeded in growth media at approximately 1 × 10^6^ cells in 10 cm cell culture dishes. After 24 h, the growth media was replaced with differentiation media consisting of DMEM supplemented with 1% FBS, 2 mM L‐glutamine, 1% penicillin–streptomycin, and 10 nM *all‐trans* retinoic acid (Sigma‐Aldrich). The cells were differentiated for 7 days, as previously described [30]. On day 7, differentiation media was removed, and the cells were washed and treated with 0.0 (vehicle control), 0.1, 0.5, 1.0, 5.0, and 10.0 mM of carnosine (Thermo Fisher Scientific, UK) or β‐alanine (Thermo Fisher Scientific, UK) for 4, 24 and 72 h. Following treatment, the culture media was collected, and the cells were lysed in 0.5% Triton X‐100 in 0.2 mM NaCl. Both homogenized cell samples and culture media samples were added to a 5% 5‐sulphosalicylic acid solution (Sigma‐Aldrich) with an internal standard of 500 μM Norleucine (Thermo Fisher Scientific, UK). The samples were incubated on ice for 30 min and then centrifuged at 10000 rpm at 4°C for 5 min. The supernatant was collected, filtered through a 0.22 μm centrifugal filter tube and stored at −80°C until analysis. Before analysis, the samples were thawed on ice and transferred to an autosampler vial (Restek, USA). The column was maintained at 50°C, and the fluorescence detector was set at an excitation wavelength of 340 nm and an emission wavelength of 450 nm. The EZ Chrom Elite Software (Agilent Technologies, USA) was used to determine the peak area. All analyte peak areas were normalized to the internal standard of Norleucine. Normalized peak areas (calculated as analyte peak area divided by internal standard peak area) were used for relative quantification.

#### 2.2.3 Real‐Time Quantitative Polymerase Chain Reaction (RT‐qPCR)

RT‐qPCR was used to determine the expression of genes related to carnosine and β‐alanine metabolism: *CARNS1, CNDP1, CNDP2, TAUT, PAT1, PEPT, PEPT2, PHT1, PHT2, GABA‐T*, and *AGXT2*. The reference gene used was 18S. Primer sequences are described in Table 1. After 7 days of cell differentiation, the media was removed, the cells were washed with phosphate‐buffered saline and then treated with 0.0 (vehicle control), 0.1, 0.5, 1.0, 5.0, and 10.0 mM of carnosine or β‐alanine for 4 h to capture acute transcriptional responses to stimuli. This time point was selected based upon previous literature that has reported transcriptional responses to β‐alanine and carnosine exposure within 1–6 h [34, 35]. Following treatment, total RNA was isolated using the phenol (TRI reagent, Invitrogen, Thermo Fisher Scientific, UK), chloroform, and alcohol method. RNA concentration and purity were measured using a micro‐spectrophotometer (NanoDrop 2000C, Thermo Fisher Scientific, UK). A 10 μL volume containing 1 μg of RNA was used to synthesize cDNA with a High‐Capacity cDNA Reverse Transcription Kit (Thermo Fisher Scientific, UK). Reverse transcription was carried out at 25°C for 37 s, 37°C for 120 min, and 85°C for 5 s.

**TABLE 1:** Forward and reverse primer sets used in the RT‐qPCR analysis of H9c2 cells.

Gene	Forward	Reverse	References
*CARNS1*	GCGGCGTCAGCAAGAAGTT	CACCAAGCAGTCATCCCAGAA	Barca et al. [31]
*CNDP1*	CCTAGAAGAATACCAGAAGAGC	GGGACTAGACGGATTGAAA	Barca et al. [31]
*CNDP2*	TTCAAGGTGTACATGGGC	AAAGGTCAAGGTCACAGGA	Barca et al. [31]
*TAUT*	TGGCCGACAGCATTCCA	GCCTTCTCTAAGGTGCCTTCCT	Everaert et al. [32]
*PAT1*	TGGTTGTACCAGTCGGTGAA	GGCCAGAACACATGTCACAC	Broberg et al. [33]
*PEPT1*	TGTGTTTGTCCTCGGCAGTGG	CTTTAGCCCAGTCCAGCCAGT	Barca et al. [31]
*PEPT2*	CATGAAATCTGTGCTCCAGG	AGGAGGCAGGAAAACAAAA	Barca et al. [31]
*PHT1*	TGAAGGCCTTGGAGTCTTT	TTGGAAATACACTGTCCAGTAA	Barca et al. [31]
*PHT2*	CCAGATGCAGTCCACCTA	AACATTGGCCAGCAGGAG	Barca et al. [31]
*GABA‐T*	CCTTCATGGGTGCTTTCCA	CAAAGGAAGGGATGTCAATCTTG	Everaert et al. [32]
*AGXT2*	GATAGGCTGCCAATCAACAATGT	TGCACTGGAGAATCTCGACAA	Blancquaert et al. [14]

RT‐qPCR was performed in duplicate with 1 μL of cDNA (25 ng), 10 μL of FastGene 2× IC Green Universal qPCR Mix low ROX (Geneflow, UK), 0.2 μL of a 20 μM forward and reverse primer mix for each gene, and 8.8 μL of nuclease‐free water. The cycling conditions were “hold” at 95°C for 2 min, 40 “cycles” of 95°C for 5 s and 60°C for 30 s, and “melt” consisting of a curve from 95°C for 5 s, 60°C for 1 min and 95°C for 15 s. Signal intensity was monitored using the QuantStudio 7 Flex Real‐Time PCR System (Applied Biosystems, UK). Results were analyzed using the comparative cycle threshold (Ct) method. Delta‐Ct (ΔCt) values were calculated for each sample and gene of interest using the formula: Ct (gene of interest) – Ct (reference gene). Relative expression levels were calculated using the 2^‐ΔΔCCt^ method [36].

### 2.3 Postmortem Human Heart

Samples from segments of the ventricular wall of sixteen individuals (mean [SD], age: 54 ± 22 years, body mass index: 23.9 ± 3.7 kg/m^2^), deceased from various causes, were donated and used in this study (Table 2). The samples were stored in a biorepository of the University of São Paulo in the vapor phase of liquid nitrogen. A small aliquot was transferred to our laboratory, where it was lyophilised for 16 h in a bench‐top freezer drier (Edwards Vacuum) and processed for β‐alanine and carnosine analysis, as well as for the qualitative analysis of the expression of selected proteins involved in carnosine metabolism, as described below. The experiment was approved by the Research Ethics Committee of the University of São Paulo (#4802079).

**TABLE 2:** Descriptive characteristics of individuals.

ID	Age (years)	Sex	Body mass (kg)	BMI (kg/m^2^)	Cause of death
1	38	M	76	23.5	Cardiac failure and bacterial pneumonia
2	68	M	80	28.3	Cardiac failure and mesenteric ischemia
3	63	M	66	22.8	Cardiac failure and bacterial pneumonia
4	13	F	*NA*	*NA*	Acute Respiratory Failure
5	48	M	74	25.9	Malignant Neoplasm
6	11	F	38	*NA*	Acute Respiratory Failure
7	66	M	88	29.4	Pneumonia and septic shock
8	60	M	59	20.2	Cerebral vascular accident
9	44	M	54	21.1	Perforated duodenal ulcer
10	62	F	54	21.4	Acute Respiratory Failure
11	54	M	71	20.7	Pneumonia and septic shock
12	78	M	48	19.7	Malignant Neoplasm
13	77	F	60	22.6	Pneumonia and septic shock
14	23	F	NA	NA	NA
15	74	F	56	23.9	Pneumonia and septic shock
16	77	M	90	31.1	Arterial occlusion

Abbreviations: BMI, body mass index; NA, not available.

#### 2.3.1 Quantification of Carnosine and β‐Alanine by HPLC‐ESI ^+^‐MS/MS

Carnosine and β‐alanine were quantified in duplicate by online HPLC‐ESI^+^‐MS/MS using CAR‐d4 as an internal standard [18, 37]. Approximately 5 mg of lyophilised tissue was powdered and deproteinised with 0.5 M HClO_4_, vortexed for 15 min and centrifuged at 5000 × *g* at 4°C for 3 min [38]. Samples were neutralized with 2.1 M KHCO_3_, centrifuged at 5000 × *g* at 4°C for 3 min, and the supernatant was used for analysis. Carnosine and β‐alanine were analyzed by electrospray ionization (ESI) in the positive mode, and detection was made using selected reaction monitoring (SRM) on a triple quadrupole mass spectrometer API 6500 (Sciex, Washington, DC, WA). An Agilent HPLC system (Agilent Technologies, Santa Clara, CA) equipped with an autosampler (1200 High Performance), a column oven set at 45°C (1200 G1216B), an automated high‐pressure flow switching valve, a 1200 Binary Pump SL, and a Shimadzu 10‐AVp Isocratic Pump (Shimadzu, Tokyo, Japan) was used for sample injection and cleanup. Separation was performed on a Kinetex C18 column (100 × 4.6 mm i.d., and 2.6 μm particle size; Phenomenex, Torrance, CA, USA), followed by a second Kinetex C18 column (100 × 2.1 mm i.d., 2.6 μm particle size; Phenomenex, Torrance, CA, USA). The mobile phase consisted of 5 mM ammonium acetate pH 5.5 (A) and acetonitrile (B). Both solutions were filtered through a 0.22 μm PVDF membrane (Millipore, Bedford, MA) before use. Separation conditions were: 0 to 4 min, 10% acetonitrile at 150 μL/min; 4–6 min, 10%–25.5% acetonitrile at 200 μL/min; 6–10 min, 30% acetonitrile at 200 μL/min; 10–15 min 30%–90% acetonitrile with flow rate increasing 200–250 μL/min; 15–20 min, 90% acetonitrile at 300 μL/min; 20–22 min, 90%–10% acetonitrile with flow rate decreasing to 200 μL/min, and then 10% acetonitrile at 150 μL/min until 32 min to allow the reequilibration of the first column. A high‐pressure flow switching valve (2‐position, 6‐port) was inserted between the two columns. The valve discarded the eluent from the first column until 3 min of run while keeping the second column supplied with a solution of ammonium acetate 5 mM: acetonitrile (9:1, v/v) at a constant flow of 150 μL/min using a Shimadzu 10‐AVp Isocratic Pump. After 3 min of run, the valve switched position, allowing the eluent from the first column to enter the second column. Upon elution from the second column, the samples were injected into the mass spectrometer. After 18 min of run, the valve switched back to the initial position, allowing the column to re‐equilibrate. The Turbo Ionspray Voltage was kept at 5500 V, the curtain gas at 20 psi, and the nebulizer and auxiliary gas at 40 psi. The temperature was set to 500°C, and the pressure of nitrogen in the collision cell was adjusted to medium. The signal‐to‐noise ratio (S/N) ≥ 7 was used as the quantification criterion. Transitions for β‐alanine were: quantification transition (m/z); confirmation transition (m/z) (90 → 72; 90 → 45 and 90 → 30). Transitions for carnosine were: quantification transition (m/z); confirmation transition (m/z) (227 → 110; 227 → 156). Transitions for CARd4 were (quantification: 231 → 110; confirmation: 231 → 156).

#### 2.3.2 Western Blot

A qualitative assessment of protein expression was conducted in *postmortem* human heart samples. Tissue samples were homogenized and extracted in RIPA buffer containing 1% protease inhibitor cocktail (Thermo Scientific). Total extracted protein was quantified using the Bradford protein assay M (BioRad), and 20 μg of protein was loaded into a polyacrylamide gel for electrophoresis. After separation, proteins were transferred to a nitrocellulose membrane using a transfer buffer containing 10% methanol. Membranes were stained with ponceau to confirm transfer efficiency and for loading control (see Supporting Information S2). Membranes were then blocked with 5% skimmed milk and incubated overnight with primary antibodies diluted in 5% skimmed milk at the following concentrations: CARSN1 (1:200; Cat# ab167240, Abcam; RRID:AB_3719947), CNDP2 (1:1000; Cat# ab241126, Abcam, RRID:AB_3719948), TauT (1:1000; Cat# ab196821, Abcam, RRID:AB_3719949), PAT1 (1:1000; Cat# ab172684, Abcam, RRID:AB_3719950), PHT1 (1:1000; Cat# LS‐C110861, LSBio, RRID:AB_10662532), PHT2 (1:1000; Cat# LS‐C161135, LSBio, RRID:AB_3719951). Membranes were then incubated with secondary antibodies (goat anti‐rabbit polyclonal, 1:5000; anti‐mouse 1:2000; Cell Signaling Technology) for 1 h. Protein bands were visualized using ECL Western Blotting Substrate (Thermo Fisher Scientific) in a C‐DiGit Blot Scanner (LI‐COR Biosciences). No densiometric quantification was performed as variability in post‐mortem tissue integrity and potential protein degradation may confound quantitative comparisons of Western Blot band intensity. Accordingly, the analysis was limited to a qualitative assessment of protein presence or absence across samples.

#### 2.3.3 Statistical Analysis

Analysis of variance (ANOVA) was performed with Graphpad Software Inc. Prism version 9.2.0 (RRID:SCR_002798) to analyze H9c2 cell data. Statistical tests were selected based on normality of the data; mainly a one‐way ANOVA with Dunnett's multiple comparison post‐test was used to compare each carnosine and β‐alanine concentration with the vehicle control for both gene expression and amino acid analyses. This could not be completed where the metabolite could not be detected in vehicle control samples. Gene expression data are presented as fold change, with statistical analysis being conducted on the ΔCt data. Differences in carnosine versus β‐alanine in human cardiac tissue were compared using a paired sample *t*‐test (R Studio, v.4.0.3, RRID:SCR_000432). Unpaired *t*‐tests with Welch's correction were used to compare differences in human cardiac carnosine and β‐alanine content between males and females. Associations between variables were explored using Pearson's correlation tests. All data are presented as mean ± 1 SD and the alpha level was set a priori at 5%.

## 3 Results

### 3.1 Acute Carnosine Incubation Increases Intracellular Carnosine in H9c2 Cardiomyocytes

**FIGURE 1:**
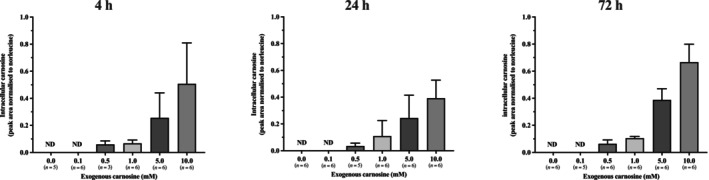
Intracellular carnosine in differentiated H9c2 cells after 4, 24, and 72 h of exogenous carnosine incubation. Data are calculated as analyte peak area normalized to the internal standard (Norleucine) peak area and presented as mean ± 1 SD. Replicate numbers included in analysis are presented underneath each respective concentration. Abbreviations: ND, not detected.

**FIGURE 2:**
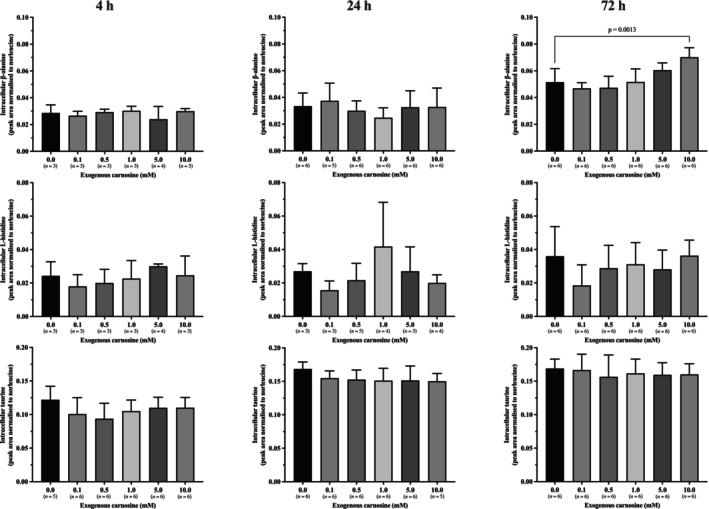
Intracellular amino acid levels (β‐alanine, L‐histidine, and taurine) in differentiated H9c2 cells after 4, 24, and 72 h of exogenous carnosine incubation. Data are calculated as analyte peak area normalized to the internal standard (Norleucine) peak area and presented as mean ± 1 SD. Replicate numbers included in analysis are presented underneath each respective concentration.

Intracellular carnosine was not detected in all vehicle control samples, but increased following the exposure to exogenous carnosine, irrespective of concentration and exposure time, except for 0.1 mM, which was not detected (Figure 1). Incubation with 10.0 mM carnosine for 72 h increased intracellular β‐alanine levels (*p* = 0.0013 vs. vehicle control), with all other conditions not being significantly different from control (all *p* > 0.05, Figure 2). No changes in intracellular taurine and L‐histidine were shown following carnosine incubation, irrespective of concentration and exposure time (all *p* > 0.05 vs. vehicle control, Figure 2).

**FIGURE 3:**
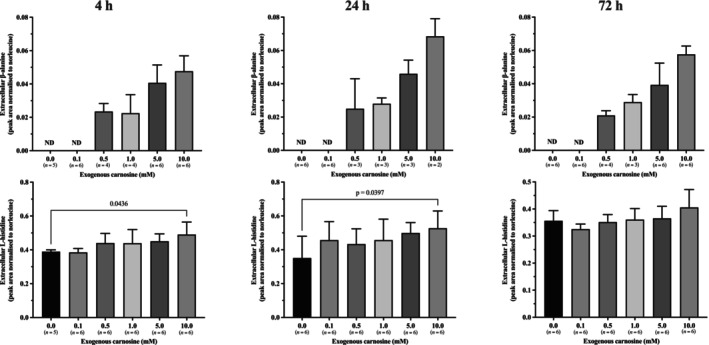
Extracellular levels of the carnosine synthesis precursors, β‐alanine and L‐histidine, in the incubation medium of differentiated H9c2 cells after 4, 24, and 72 h of exogenous carnosine incubation. Data are calculated as analyte peak area normalized to the internal standard (Norleucine) peak area and presented as mean ± 1 SD. Replicate numbers included in analysis are presented underneath each respective concentration. Abbreviations: ND, not detected.

We measured extracellular amino acid concentrations to investigate whether carnosine degradation occurred in the medium when H9c2 cardiomyocytes were incubated with acute carnosine. This confirmed an increase in free β‐alanine in all concentrations in all exposure times, except 0.1 mM. L‐histidine was only increased with 10.0 mM of exogenous carnosine at 4 and 24 h (*p* = 0.0436 and *p* = 0.0397 vs. vehicle control for 4 and 24 h) (Figure 3).

Intra‐ and extracellular anserine were not detected in all samples (*n* = 6). No changes in extracellular taurine were shown following carnosine incubation for 4 h (*data not shown*), irrespective of concentration (all *p* > 0.05 vs. vehicle control), but quantification of taurine in the incubation medium of the H9c2 cells was not possible (not detected) in almost all samples following 24 and 72 h.

### 3.2 Acute β‐Alanine Incubation Increases Intracellular β‐Alanine Concentrations in H9c2 Cardiomyocytes

**FIGURE 4:**
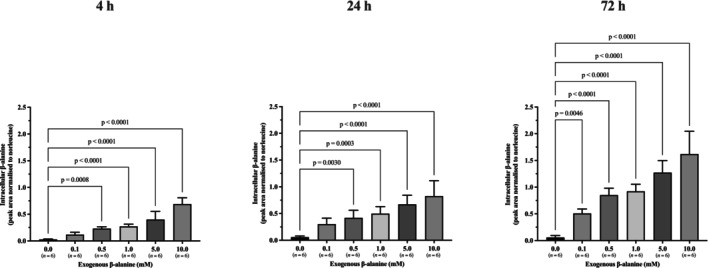
Intracellular β‐alanine in differentiated H9c2 cells after 4, 24, and 72 h of exogenous β‐alanine incubation. Data are calculated as analyte peak area normalized to the internal standard (Norleucine) peak area and presented as mean ± 1 SD. Replicate numbers included in analysis are presented underneath each respective concentration.

Intracellular β‐alanine increased in H9c2 cardiomyocytes following incubation with β‐alanine at all exposure times and concentrations (except for 0.1 mM of β‐alanine at 4 and 24 h, *p* > 0.05) (Figure 4).

**FIGURE 5:**
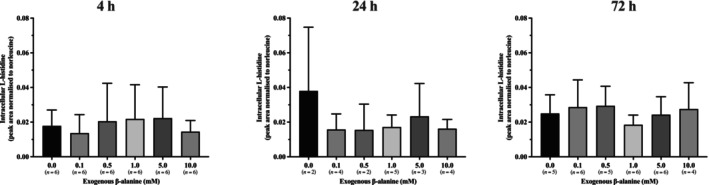
Intracellular L‐histidine in differentiated H9c2 cells after 4, 24, and 72 h of exogenous β‐alanine incubation. Data are calculated as analyte peak area normalized to the internal standard (Norleucine) peak area and presented as mean ± 1 SD. Replicate numbers included in analysis are presented underneath each respective concentration.

Incubation with β‐alanine, irrespective of concentration and exposure time, did not increase intracellular HCD levels; intracellular carnosine and anserine were not detected in all samples (*n* = 6). Intracellular L‐histidine also remained unchanged with exposure to β‐alanine (*p* > 0.05 vs. vehicle control for all concentrations and time points) (Figure 5).

**FIGURE 6:**
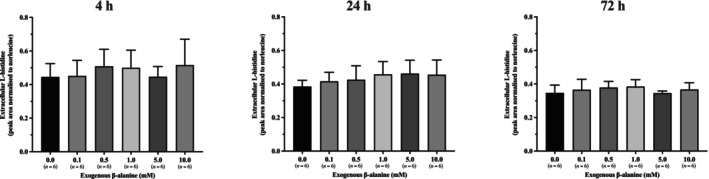
Extracellular L‐histidine in the incubation medium of differentiated H9c2 cells after 4, 24, and 72 h of exogenous β‐alanine incubation. Data are calculated as analyte peak area normalized to the internal standard (Norleucine) peak area and presented as mean ± 1 SD. Replicate numbers included in analysis are presented underneath each respective concentration.

Exposure to β‐alanine, irrespective of concentration and time, did not change the levels of L‐histidine (all *p* > 0.05 vs. vehicle control, Figure 6), carnosine, or anserine in the incubation medium. Carnosine and anserine in the medium were not detected in all extracellular samples (*n* = 6).

### 3.3 Acute β‐Alanine Incubation Drives Intracellular Taurine Depletion and Extracellular Taurine Accumulation in H9c2 Cardiomyocytes

**FIGURE 7:**
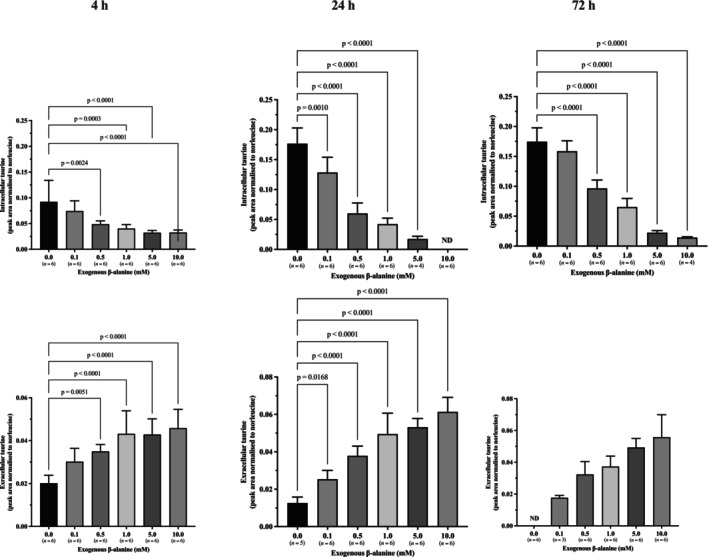
Intra‐ and extracellular taurine in differentiated H9c2 cardiomyocytes after 4, 24, and 72 h of exogenous β‐alanine incubation. Data are calculated as analyte peak area normalized to the internal standard (Norleucine) peak area and presented as mean ± 1 SD. Replicate numbers included in analysis are presented underneath each respective concentration. Abbreviations: ND, not detected.

Intracellular taurine significantly decreased following β‐alanine exposure at all times and concentrations (all *p* < 0.05 vs. vehicle control), except 0.1 mM at 4 and 72 h (4 h, *p* = 0.3754; 72 h, *p* = 0.2470) and taurine was not detected when cells were incubated with 10.0 mM of β‐alanine for 24 h (Figure 7). In parallel, extracellular taurine increased following β‐alanine incubation for 4 and 24 h, with all concentrations being significantly different from the vehicle control (all *p* > 0.05), except 0.1 mM following 4 h (*p* = 0.0836) (Figure 7). For 72 h, taurine could not be detected in the incubation medium in vehicle control samples. When H9c2 cells were exposed to the various β‐alanine concentrations for 72 h, however, taurine could be detected in the incubation medium (Figure 7).

### 3.4 H9c2 Cardiomyocytes Express Carnosine‐Related Enzymes and Transporters

The H9c2 cardiomyocytes expressed *CARNS1, CNDP1, CNDP2, TAUT, PAT1, PEPT1, PEPT2, PHT1, PHT2*, and *GABA‐T* genes, whilst *AGXT2* expression was not detected.

### 3.5 Acute Carnosine Incubation Did Not Affect Carnosine‐Related Enzyme and Transporter Gene Expressions

**FIGURE 8:**
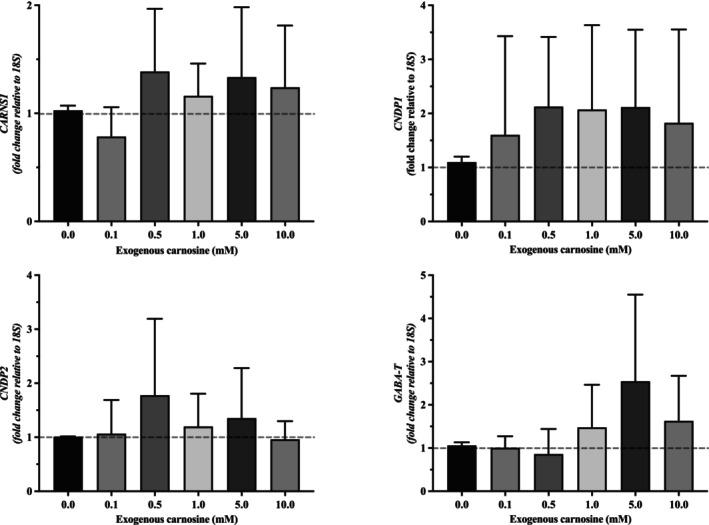
Gene expression of enzymes involved in carnosine synthesis (*CARNS1*) and hydrolysis (*CNDP1* and *CNDP2*) and β‐alanine transamination (*GABA‐T*) in differentiated H9c2 cardiomyocyte cells after 4 h of exogenous carnosine incubation. Data are presented as fold change, normalized to the reference gene *18S*, with statistical analyses conducted on delta Ct values. All concentrations have *n* = 6, except for 0.1 mM (*n* = 4) and 0.5 mM (*n* = 5). Data are presented as mean ± 1 SD.

**FIGURE 9:**
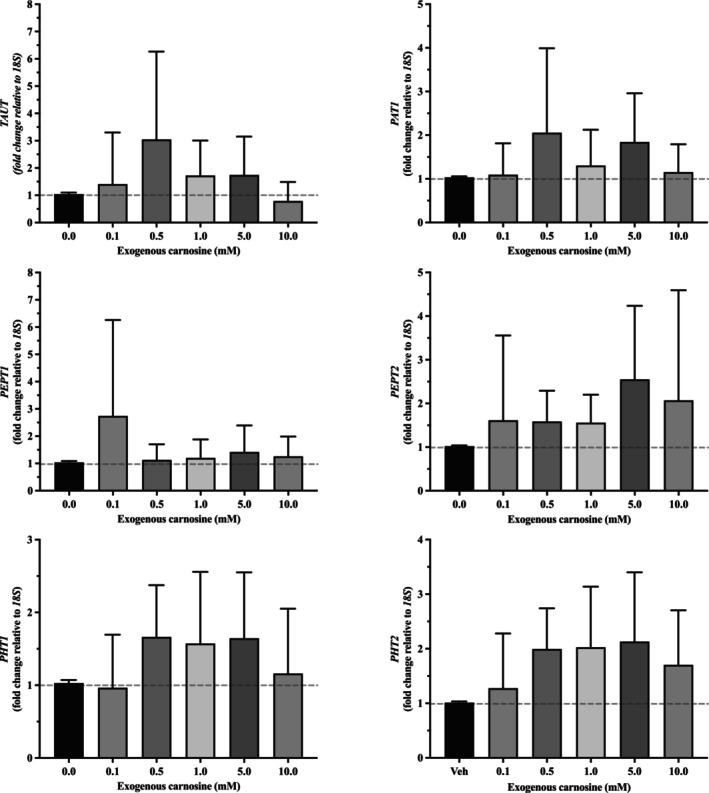
Gene expression of amino acid transporters (*TAUT* and *PAT1*) and non‐specific peptide transporters (*PEPT1, PEPT2, PHT1* and *PHT2*) in differentiated H9c2 cardiomyocytes after 4 h of exogenous carnosine incubation. Data are presented as fold change, normalized to the reference gene *18S*, with statistical analyses conducted on delta Ct values. All concentrations have *n* = 6, except for 0.1 mM (*n* = 4) and 0.5 mM (*n* = 5). Data are presented as mean ± 1 SD.

Treatment with 0.1, 0.5, 1, 5.0, and 10.0 mM of carnosine for 4 h did not alter the expression of *CARNS1, CNDP1*, and *CNDP2* (Figure 8), the amino acid transporters, *TAUT* and *PAT1* (Figure 9), the β‐alanine transaminase enzyme, *GABA‐T* (Figure 8), or the non‐specific peptide transporters, *PEPT1, PEPT2, PHT1*, and *PHT2* (Figure 9) (all *p* > 0.05 vs. vehicle control).

### 3.6 Acute β‐Alanine Incubation Downregulates TAUT and PHT1 Gene Expression

**FIGURE 10:**
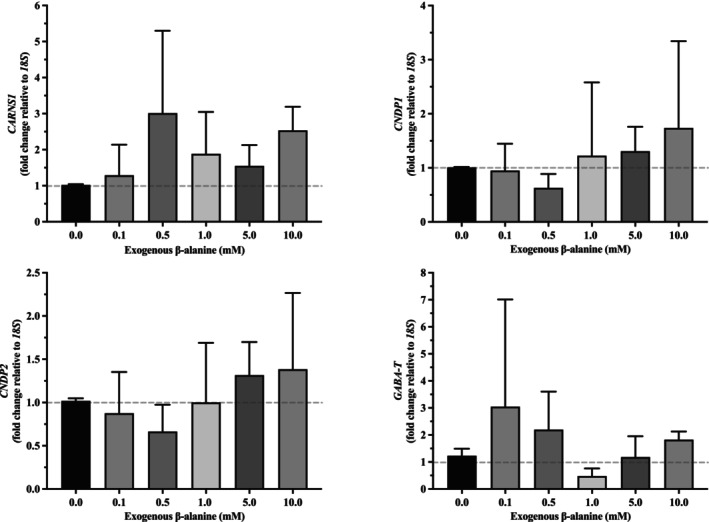
Gene expression of enzymes involved in carnosine synthesis (*CARNS1*) and hydrolysis (*CNDP1* and *CNDP2*) and β‐alanine transamination (*GABA‐T*) in differentiated H9c2 cardiomyocyte cells after 4 h of exogenous β‐alanine incubation. Data are presented as fold change, normalized to the reference gene *18S*, with statistical analyses conducted on delta Ct values. Biological replicates range between three to six: 0.0 mM, *n* = 6; 0.1 mM, *n =* 4; 0.5 mM, *n* = 3; 1.0 mM, *n =* 4; 5.0 mM, *n* = 5; 10.0 mM, *n* = 4. Data are presented as mean ± 1 SD.

**FIGURE 11:**
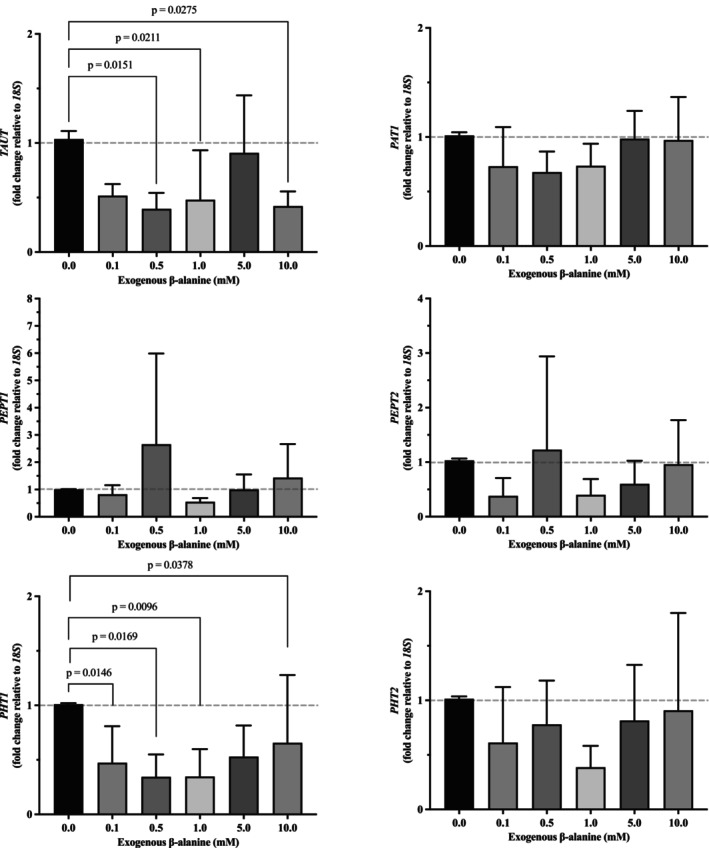
Gene expression of amino acid transporters (*TAUT* and *PAT1*) and non‐specific peptide transporters (*PEPT1, PEPT2, PHT1* and *PHT2*) in differentiated H9c2 cardiomyocyte cells after 4 h of exogenous β‐alanine incubation. Data are presented as fold change, normalized to the reference gene *18S*, with statistical analyses conducted on delta Ct values. Biological replicates range between three to six: 0.0 mM, *n* = 6; 0.1 mM, *n =* 4; 0.5 mM, *n* = 3; 1.0 mM, *n =* 4; 5.0 mM, *n* = 5; 10.0 mM, *n* = 4. Data are presented as mean ± 1SD.

Treatment with all β‐alanine concentrations for 4 h did not affect the expression of the enzymes *CARNS1, CNDP1*, and *CNDP2* (Figure 10), the amino acid transporter *PAT1* (Figure 11), the β‐alanine transaminase *GABA‐T* (Figure 10), or the non‐specific peptide transporters *PEPT1, PEPT2*, *and PHT2* (Figure 11) (all *p* > 0.05).

The administration of exogenous β‐alanine for 4 h significantly decreased the expression of *TAUT* at 0.5 mM (−62%, *p* = 0.0151), 1.0 mM (−54%, *p* = 0.0211) and 10.0 mM (−59%, *p* = 0.0275) compared to vehicle control, but no differences from control were shown at 0.1 mM (−50%, *p* = 0.1021) and 5.0 mM (−12%, *p* = 0.5966). *PHT1* expression was significantly decreased at all β‐alanine concentrations (−35% to −66%, *p* < 0.05) except at 5.0 mM (−48%, *p* = 0.0658) when compared to the vehicle control (Figure 11).

### 3.7 Carnosine, β‐Alanine, and Carnosine‐Related Enzymes and Transporters Are Present in Human Heart

**FIGURE 12:**
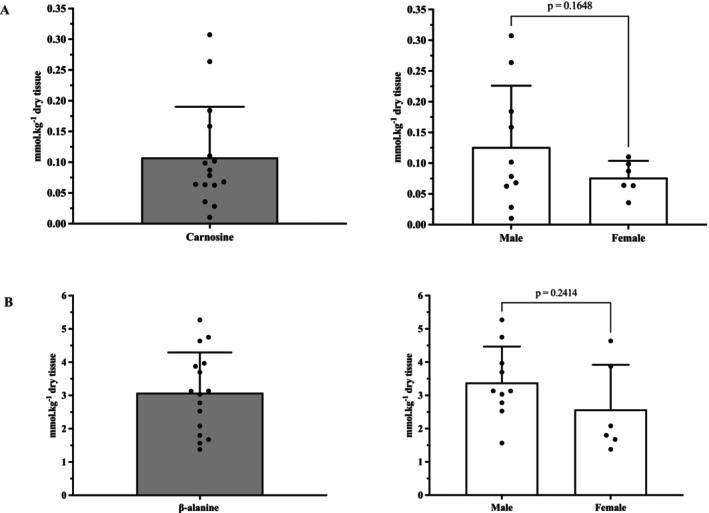
Carnosine (A) and β‐alanine (B) content in heart samples from all individuals grouped and separated by sex. Data are presented as mean ± 1 SD.

**FIGURE 13:**
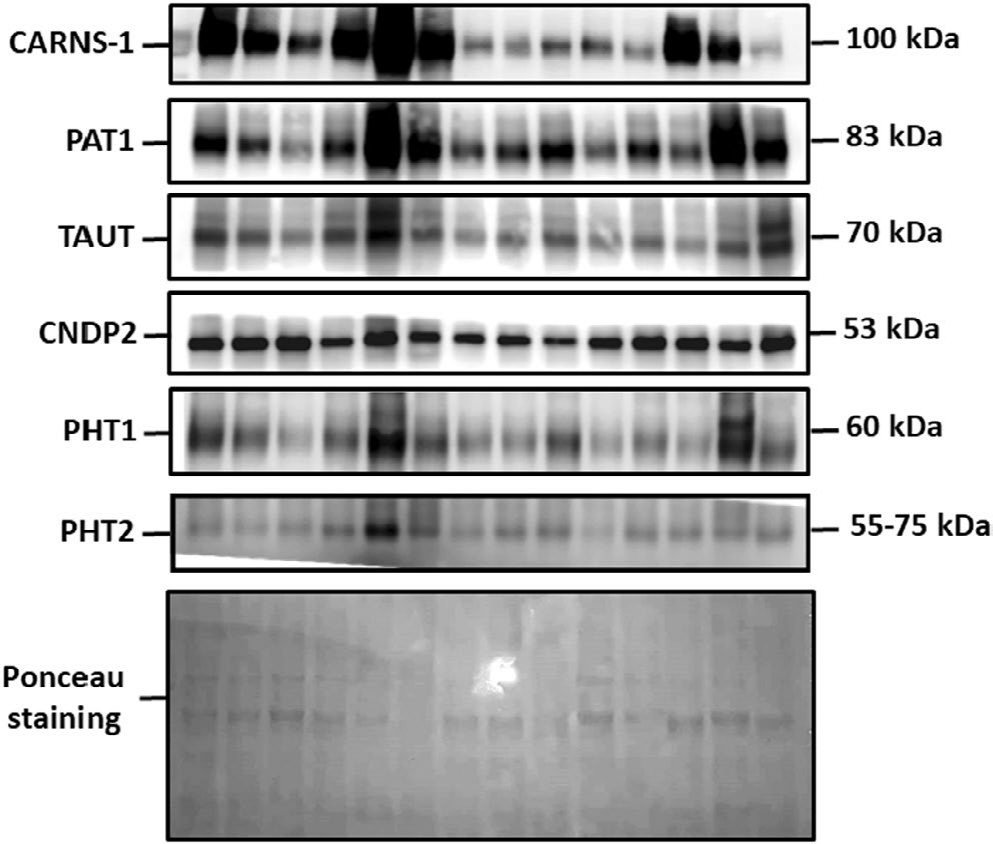
Images of the immunoblotting membranes depicting the expression of carnosine‐related metabolism proteins in 14 human heart samples. Each lane contains a sample of a single participant, except for the first lane on the left, which contains the protein ladder. Ponceau S staining of the membranes is included to verify equal protein loading and transfer.

Both carnosine and β‐alanine were detected in human heart samples within the low millimolar range, with β‐alanine values being ~30 times higher than carnosine (*p* < 0.0001; Figure 12). Carnosine and β‐alanine contents displayed large interindividual variation (0.01–0.31 mmol·kg^−1^ dry muscle for carnosine, and ~1–5 mmol·kg^−1^ dry muscle for β‐alanine). No significant differences were shown between males and females for carnosine (*p* = 0.1648) and β‐alanine (*p* = 0.2414) content. Neither carnosine nor β‐alanine in heart were significantly correlated with age (*r* = −0.008, *p* = 0.98; *r* = 0.344, *p* = 0.19). The immunoblotting analysis confirmed the presence of CARNS1, CNDP2, TAUT, PAT1, PHT1 and PHT2 in human ventricular wall (Figure 13).

## 4 Discussion

Rat ventricular cardiomyocytes (H9c2 cells) expressed the genes for the enzymes and transporters involved in carnosine and β‐alanine metabolism, with protein expression confirmed in samples taken from the human ventricle. Further analysis indicated that H9c2 cells were capable of taking up and accumulating exogenous sources of β‐alanine and carnosine, although increased intracellular β‐alanine did not result in the synthesis of carnosine or anserine within a 72 h timeframe. This indicates that exogenous β‐alanine can be taken up by cardiomyocytes, providing support for future intervention trials to be conducted looking at the direct effect on cardiac physiology.

β‐alanine supplementation is commonly used as a model of taurine deficiency [39, 40, 41, 42, 43] as the amino acids share the sodium/chloride dependent transporter, TAUT, and may compete for this in a time‐dependent manner [44]. In H9c2 cardiomyocytes, the intracellular concentration of taurine decreased with the provision of exogenous β‐alanine; this was coupled with an increase in the concentration of extracellular taurine, as shown in the culture media. The decrease of taurine may be due to exogenous β‐alanine limiting the uptake of taurine from the culture media into the cells (taurine is present in the FBS in the media), or taurine being exported into the media from the cells when β‐alanine is taken up. In H9c2 cardiomyocytes, exogenous β‐alanine decreased the expression of *TAUT*, which may also explain the decrease of taurine in the cells when incubated with β‐alanine. The practical implications of the interaction between these two amino acids remain unclear, with the literature showing detrimental [40, 41, 42], beneficial [39], and no effects [43] of taurine depletion on myocardial function and health. Although direct measurements of taurine in human models are not feasible due to the ethical constraints of invasive cardiac sampling, 12 weeks of 4.8 g/day of β‐alanine supplementation did not alter cardiac structure or function in people with overweight or obesity [45].

The H9c2 study demonstrated an increase in intracellular carnosine in cardiomyocytes when the cells are supplemented with carnosine, but only with high concentrations; this may be due to the low efficiency of the carnosine transporter, meaning that a higher concentration of carnosine is required for the transporter to more efficiently take carnosine up the cells. As the cells were exposed to exogenous carnosine in the media, there was a subsequent increase in β‐alanine in the culture media (extracellular). Both growth and differentiation media contain FBS, which might suggest the presence of serum carnosinase, which would hydrolyse the exogenous carnosine into its constituent amino acids. The increase in extracellular β‐alanine only occurred with 5 and 10 mM of exogenous carnosine, potentially due to CNDP1 enzyme activity only being saturated at these concentrations. Due to carnosine degradation only occurring at the highest concentrations, and the lack of HCD synthesis with β‐alanine incubation, it can be assumed that the exogenous carnosine was taken into the cell in its intact form, rather than being hydrolysed into β‐alanine and further synthesized once in the cell.

Exposure to exogenous β‐alanine increased the intracellular concentration of β‐alanine in H9c2 cardiomyocytes, although there was no increase in carnosine or anserine concentrations, indicating that no measurable HCD accumulation occurred during the 72 h measurement window. L‐histidine was present in the cells, and the growth and differentiation media (0.2 mM), so it was unlikely to be the rate‐limiting factor for HCD synthesis in this experiment. It has been suggested that when ample β‐alanine is provided and the transporter, TAUT, is saturated, the activity of the enzyme (CARNS1) may be the rate‐limiting factor for the synthesis of carnosine [46]. CARNS1 is considered a “sluggish’ enzyme”, with a Kcat value of less than 1 per second [12]. The enzymatic activity is suggested to be 10 times lower in myocardial tissue when compared to skeletal muscle [47]. Thus, strategies to increase the enzymatic activity of CARNS1 are necessary [37]. The lack of anserine synthesis with β‐alanine provision is likely explained by the absence of carnosine synthesis. Anserine is a methylated analogue of carnosine, with the synthesis being dependent upon the intracellular concentration of carnosine [27]. Although intracellular carnosine levels increased with the provision of exogenous carnosine, no corresponding increase in intracellular anserine was detected. This suggests that, like CARNS1, the enzyme responsible for anserine synthesis may be the rate‐limiting factor in this reaction. Additionally, the experiment was limited by the timeframe in which the H9c2 cells could be incubated with the exogenous source. It is also possible that a lack of detectable HCD accumulation following β‐alanine provision was due to limitations in the sensitivity of the detection method, which may not have been capable of detecting very small changes. Future studies need to measure the activity of these enzymes to begin to understand whether this is the limiting factor for HCD synthesis in cardiomyocytes when ample exogenous β‐alanine is available.

There are discrepancies in the literature regarding HCD concentrations in cardiac tissue [48], with a likely explanation being differences in accuracy and sensitivity between different analytical techniques [1]. The use of HPLC‐MS/MS with high sensitivity and accuracy in human heart samples is a refinement of the literature in that regard. Endogenous carnosine and β‐alanine were detected in human ventricular heart samples. The presence of endogenous β‐alanine may be an artifact resulting from the degradation of the sample from tissue collection to the public morgue system. Human heart samples expressed the protein for key enzymes and transporters involved in carnosine and β‐alanine metabolism, indicating the molecular machinery is present there to transport and uptake carnosine and β‐alanine supplementation. There was large inter‐individual variation between tissue samples, which is likely attributed to the heterogeneity in donor characteristics, such as health conditions, particularly the presence of heart disease, which may deplete local carnosine by forming adducts [28, 49]. Another potential source of variation is the *post‐mortem* sample processing time; although this factor could not be controlled. Interestingly, although age and sex—both factors known to influence carnosine content in skeletal muscle [50]—did not appear to be associated with differences in cardiac carnosine and β‐alanine, this individual variation warrants further investigation.

The study has several limitations including the use of cells derived from rat ventricular cardiomyocytes, with translation of these results regarding the viability of in vivo supplementation needing to be approached with caution. In addition, transcriptional analyses were restricted to a single early time point (4 h) to explore acute gene expression responses to β‐alanine and carnosine exposure, rather than longer‐term transcriptional remodeling. As changes in gene expression do not necessarily reflect enzyme activity or intracellular metabolite concentrations, and carnosine accumulation is largely driven by substrate availability [14, 32], the experiment prioritized an extended metabolite time‐course (up to 72 h) to capture cumulative concentration changes. Consequently, sustained or delayed transcriptional adaptations may not have been detected. The human heart ventricle samples were only analyzed for carnosine and β‐alanine, whilst the presence of anserine had been confirmed previously [11]. Other HCDs that may be present in the heart include homocarnosine and N‐acetylcarnosine; however, we were unable to quantify these peptides. Alongside the data by Van der Stede et al. [3], it can be suggested that carnosine and anserine are the predominant HCDs present in human heart. The human samples in both our study and Van der Stede et al. [3], however, were collected from “diseased” patients; different pathologies, with different metabolic changes, may influence HCD metabolism and content. Lastly, the human samples used in this study must be considered unsuitable for quantitative analysis due to unknown *postmortem* processing time. Thus, caution should be exercised when interpreting the absolute values for dipeptide concentrations; this also precludes us from performing a quantitative approach to tissue protein expression. Nonetheless, our data clearly show that both the dipeptides and their enzymatic machinery are expressed in human cardiac tissue.

## 5 Conclusion

H9c2 rat ventricular cardiomyocytes and human heart ventricle samples expressed the enzymes and transporters required for carnosine and β‐alanine metabolism. The H9c2 cells demonstrated that exogenous carnosine and β‐alanine could be taken up and accumulated in their intact form, indicating potential viability for in vivo supplementation studies.

## Author Contributions

Jade V. Creighton: conceived and designed research; performed experiments; analyzed data; interpreted results of experiments; prepared figures; drafted manuscript; edited and revised manuscript; approved final version of manuscript. Lívia de Souza Gonçalves: conceived and designed research; performed experiments; analyzed data; prepared figures; drafted manuscript; edited and revised manuscript; approved final version of manuscript. Saulo Gil: performed experiments; analyzed data; prepared figures; approved final version. Bianca Scigliano Vargas: performed experiments; analyzed data; prepared figures; interpreted results of the experiments; approved final version. Leonardo Jensen: performed experiments; analyzed data; prepared figures; interpreted results of the experiments; approved final version. Marisa Helena Gennari de Medeiros: conceived and designed research; analyzed data; interpreted results of the experiments; edited and revised manuscript; approved final version. Hamilton Roschel: conceived and designed research; interpreted results of the experiments; edited and revised manuscript; approved final version. Mark D. Turner: conceived and designed research; edited and revised manuscript; approved final version of manuscript. Craig L. Doig: conceived and designed research; edited and revised manuscript; approved final version of manuscript. Guilherme Giannini Artioli: conceived and designed research; analyzed data; interpreted results of the experiments; prepared figures; edited and revised manuscript; approved final version. Craig Sale: conceived and designed research; edited and revised manuscript; approved final version of manuscript. Jade V. Creighton and Lívia de Souza Gonçalves should be considered joint first authors.

## Funding

Jade V. Creighton was on a match‐funded PhD studentship between Nottingham Trent University and Natural Alternatives International (NAI), a company formulating and manufacturing customized nutritional supplements, including CarnoSyn *β*‐alanine. Guilherme Giannini Artioli and Hamilton Roschel were funded by Sao Paulo Research Foundation (FAPESP; grant numbers 2014/11948‐8, 2019/25032‐9, and 2024/18769‐3). Saulo Gil is supported by FAPESP (grant number 2023/15629‐3).

## Conflicts of Interest

Craig Sale is the recipient of funding to support a PhD studentship relating to the effects of carnosine on cardiac function from NAI (i.e., the funding to support Jade V. Creighton). Jade V. Creighton, Craig Sale, and Guilherme Giannini Artioli have received supplements for other studies free of charge from NAI. NAI has contributed to the payment of open‐access publication charges for some manuscripts on supplementation for Craig Sale and Guilherme Giannini Artioli. Craig Sale has also received an honorarium from NAI to produce materials to support a blog on β‐alanine supplementation and the effects of carnosine. The other authors declare no conflicts of interest.

## Supporting information


**Table SI:** Intracellular experimental replicates for 4‐hour treatment of carnosine in H9c2 cells.
**Table SII:** Extracellular experimental replicates for 4‐hour treatment of carnosine in H9c2 cells.
**Table SIII:** Intracellular experimental replicates for 24‐hour treatment of carnosine in H9c2 cells.
**Table SIV:** Extracellular experimental replicates for 24‐hour treatment of carnosine in H9c2 cells.
**Table SV:** Intracellular experimental replicates for 72‐hour treatment of carnosine in H9c2 cells.
**Table SVI:** Extracellular experimental replicates for 72‐hour treatment of carnosine in H9c2 cells.
**Table SVII:** Intracellular experimental replicates for 4‐hour treatment of β‐alanine in H9c2 cells.
**Table SVIII:** Extracellular experimental replicates for 4‐hour treatment of β‐alanine in H9c2 cells.
**Table SIX:** Intracellular experimental replicates for 24‐hour treatment of β‐alanine in H9c2 cells.
**Table SX:** Extracellular experimental replicates for 24‐hour treatment of β‐alanine in H9c2 cells.
**Table SXI:** Intracellular experimental replicates for 72‐hour treatment of β‐alanine in H9c2 cells.
**Table SXII:** Extracellular experimental replicates for 72‐hour treatment of β‐alanine in H9c2 cells.

**Figure SI:** Ponceau staining loading controls for Western Blot membranes: (A) CARNS1, (B) CNDP2, (C) TAUT, (D) PAT1, (E) PHT1, and (F) PHT2.

## Data Availability

The data that support the findings of this study are available on request from the corresponding author (Craig Sale).
