# Supplementary material for: Carnosine‐Related Metabolism in Rat Cardiomyocytes and Human Heart Tissue
Source: FASEB J. 2026 Jul 15;40(14):e72033. doi: 10.1096/fj.202504676R (PMC13371992; doi:10.1096/fj.202504676R)
Supplement: Supplementary file 1 — Table SI: Intracellular experimental replicates for 4‐hour treatment of carnosine in H9c2 cells. Table SII: Extracellular experimental replicates for 4‐hour treatment of carnosine in H9c2 cells. Table SIII: Intracellular experimental replicates for 24‐hour treatment of carnosine in H9c2 cells. Table SIV: Extracellular experimental replicates for 24‐hour treatment of carnosine in H9c2 cells. Table SV: Intracellular experimental replicates for 72‐hour treatment of carnosine in H9c2 cells. Table SVI: Extracellular experimental replicates for 72‐hour treatment of carnosine in H9c2 cells. Table SVII: Intracellular experimental replicates for 4‐hour treatment of β‐alanine in H9c2 cells. Table SVIII: Extracellular experimental replicates for 4‐hour treatment of β‐alanine in H9c2 cells. Table SIX: Intracellular experimental replicates for 24‐hour treatment of β‐alanine in H9c2 cells. Table SX: Extracellular experimental replicates for 24‐hour treatment of β‐alanine in H9c2 cells. Table SXI: Intracellular experimental replicates for 72‐hour treatment of β‐alanine in H9c2 cells. Table SXII: Extracellular experimental replicates for 72‐hour treatment of β‐alanine in H9c2 cells. [file FSB2-40-e72033-s002.docx]

Supplementary Material 1

Description: Each concentration had six experimental replicates completed, with all metabolites analysed in each replicate. Some were excluded from the final analysis due to non-detection or poor quality as specified. Each table specifies how many replicates were included in the final analysis, how many were not detected and, therefore, could not be quantified or included in final analysis, and how many replicates experienced an issue during analysis and were not included in final analysis.

| **Supplementary Table I. Intracellular experimental replicates for 4-hour treatment of carnosine in H9c2 cells.** | | | | | |
| --- | --- | --- | --- | --- | --- |
| **Carnosine concentration (mM)** | **Intracellular taurine** | **Intracellular β-alanine** | **Intracellular L-histidine** | **Intracellular carnosine** | **Intercellular anserine** |
| 0.0 | 5 replicates included  Issue with 1 replicate | 3 replicates included  2 replicates ND  Issue with 1 replicate | 3 replicates included  2 replicates ND  Issue with 1 replicate | 5 replicates ND  Issue with 1 replicate | 5 replicates ND  Issue with 1 replicate |
| 0.1 | 6 replicates included | 3 replicates included  3 replicates ND | 3 replicates included  3 replicates ND | 6 replicates ND | 6 replicates ND |
| 0.5 | 6 replicates included | 3 replicates included  3 replicates ND | 3 replicates included  3 replicates ND | 3 replicates included  3 replicates ND | 6 replicates ND |
| 1.0 | 6 replicates included | 3 replicates included  3 replicates ND | 3 replicates included  3 replicates ND | 6 replicates included | 6 replicates ND |
| 5.0 | 6 replicates included | 4 replicates included  2 replicates ND | 2 replicates included  4 replicates ND | 6 replicates included | 6 replicates ND |
| 10.0 | 6 replicates included | 3 replicates included  3 replicates ND | 3 replicates included  3 replicates ND | 6 replicates included | 6 replicates ND |
| *Abbreviations: ND, not detected.* | | | | | |

| **Supplementary Table II. Extracellular experimental replicates for 4-hour treatment of carnosine in H9c2 cells.** | | | | | |
| --- | --- | --- | --- | --- | --- |
| **Carnosine concentration (mM)** | **Extracellular taurine** | **Extracellular β-alanine** | **Extracellular L-histidine** | **Extracellular carnosine** | **Extracellular anserine** |
| 0.0 | 5 replicates included Issue with 1 replicate | 5 replicates included  Issue with 1 replicate | 5 replicates included  Issue with 1 replicate | Not analysed | 6 replicates ND |
| 0.1 | 6 replicates included | 6 replicates ND | 6 replicates included | Not analysed | 6 replicates ND |
| 0.5 | 5 replicates included  1 replicate ND | 4 replicates included  2 replicates ND | 6 replicates included | Not analysed | 6 replicates ND |
| 1.0 | 6 replicates included | 4 replicates included  2 replicates ND | 6 replicates included | Not analysed | 6 replicates ND |
| 5.0 | 6 replicates included | 6 replicates included | 6 replicates included | Not analysed | 6 replicates ND |
| 10.0 | 6 replicates included | 6 replicates included | 6 replicates included | Not analysed | 6 replicates ND |
| *Abbreviations: ND, not detected.* | | | | | |

| **Supplementary Table III. Intracellular experimental replicates for 24-hour treatment of carnosine in H9c2 cells.** | | | | | |
| --- | --- | --- | --- | --- | --- |
| **Carnosine concentration (mM)** | **Intracellular taurine** | **Intracellular β-alanine** | **Intracellular L-histidine** | **Intracellular carnosine** | **Intercellular anserine** |
| 0.0 | 6 replicates included | 6 replicates included | 3 replicates included  3 replicates ND | 6 replicates ND | 6 replicates ND |
| 0.1 | 6 replicates included | 5 replicates included  1 replicate ND | 3 replicates included  3 replicates ND | 6 replicates ND | 6 replicates ND |
| 0.5 | 6 replicates included | 6 replicates included | 5 replicates included  1 replicate ND | 5 replicates included  1 replicate ND | 6 replicates ND |
| 1.0 | 6 replicates included | 6 replicates included | 4 replicates included  2 replicates ND | 6 replicates included | 6 replicates ND |
| 5.0 | 6 replicates included | 6 replicates included | 3 replicates included  3 replicates ND | 6 replicates included | 6 replicates ND |
| 10.0 | 6 replicates included | 6 replicates included | 4 replicates included  2 replicates ND | 6 replicates included | 6 replicates ND |
| *Abbreviations: ND, not detected.* | | | | | |

| **Supplementary Table IV. Extracellular experimental replicates for 24-hour treatment of carnosine in H9c2 cells.** | | | | | |
| --- | --- | --- | --- | --- | --- |
| **Carnosine concentration (mM)** | **Extracellular taurine** | **Extracellular β-alanine** | **Extracellular L-histidine** | **Extracellular carnosine** | **Extracellular anserine** |
| 0.0 | 2 replicates included  4 replicates ND | 6 replicates ND | 6 replicates included | Not analysed | 6 replicates ND |
| 0.1 | 2 replicates included  4 replicates ND | 6 replicates ND | 6 replicates included | Not analysed | 6 replicates ND |
| 0.5 | 3 replicates included  3 replicates ND | 3 replicates included  3 replicates ND | 6 replicates included | Not analysed | 6 replicates ND |
| 1.0 | 3 replicates included  3 replicates ND | 3 replicates included  3 replicates ND | 6 replicates included | Not analysed | 6 replicates ND |
| 5.0 | 5 replicates included  1 replicate ND | 3 replicates included  3 replicates ND | 6 replicates included | Not analysed | 6 replicates ND |
| 10.0 | 5 replicates included  1 replicate ND | 2 replicates included  3 replicates ND  Issue with 1 replicate | 6 replicates included | Not analysed | 6 replicates ND |
| *Abbreviations: ND, not detected.* | | | | | |

| **Supplementary Table V. Intracellular experimental replicates for 72-hour treatment of carnosine in H9c2 cells.** | | | | | |
| --- | --- | --- | --- | --- | --- |
| **Carnosine concentration (mM)** | **Intracellular taurine** | **Intracellular β-alanine** | **Intracellular L-histidine** | **Intracellular carnosine** | **Intercellular anserine** |
| 0.0 | 6 replicates included | 6 replicates included | 6 replicates included | 6 replicates ND | 6 replicates ND |
| 0.1 | 6 replicates included | 6 replicates included | 6 replicates included | 5 replicates ND  Issue with 1 replicate | 6 replicates ND |
| 0.5 | 6 replicates included | 6 replicates included | 6 replicates included | 6 replicates included | 6 replicates ND |
| 1.0 | 6 replicates included | 6 replicates included | 6 replicates included | 6 replicates included | 6 replicates ND |
| 5.0 | 6 replicates included | 6 replicates included | 6 replicates included | 6 replicates included | 6 replicates ND |
| 10.0 | 6 replicates included | 6 replicates included | 6 replicates included | 6 replicates included | 6 replicates ND |
| *Abbreviations: ND, not detected.* | | | | | |

| **Supplementary Table VI. Extracellular experimental replicates for 72-hour treatment of carnosine in H9c2 cells.** | | | | | |
| --- | --- | --- | --- | --- | --- |
| **Carnosine concentration (mM)** | **Extracellular taurine** | **Extracellular β-alanine** | **Extracellular L-histidine** | **Extracellular carnosine** | **Extracellular anserine** |
| 0.0 | 2 replicates included  4 replicates ND | 6 replicates ND | 6 replicates included | Not analysed | 6 replicates ND |
| 0.1 | 1 replicate detected  5 replicates ND | 6 replicates ND | 6 replicates included | Not analysed | 6 replicates ND |
| 0.5 | 6 replicates ND | 4 replicates included  2 replicates ND | 6 replicates included | Not analysed | 6 replicates ND |
| 1.0 | 1 replicate detected  5 replicates ND | 3 replicates included  3 replicates ND | 6 replicates included | Not analysed | 6 replicates ND |
| 5.0 | 6 replicates ND | 6 replicates included | 6 replicates included | Not analysed | 6 replicates ND |
| 10.0 | 2 replicates detected  4 replicates ND | 6 replicates included | 6 replicates included | Not analysed | 6 replicates ND |
| *Abbreviations: ND, not detected.* | | | | | |

| **Supplementary Table VII. Intracellular experimental replicates for 4-hour treatment of β-alanine in H9c2 cells.** | | | | | |
| --- | --- | --- | --- | --- | --- |
| **β-alanine concentration (mM)** | **Intracellular taurine** | **Intracellular β-alanine** | **Intracellular L-histidine** | **Intracellular carnosine** | **Intercellular anserine** |
| 0.0 | 6 replicates included | 6 replicates included | 6 replicates included | 6 replicates ND | 6 replicates ND |
| 0.1 | 6 replicates included | 6 replicates included | 6 replicates included | 6 replicates ND | 6 replicates ND |
| 0.5 | 6 replicates included | 6 replicates included | 6 replicates included | 6 replicates ND | 6 replicates ND |
| 1.0 | 6 replicates included | 6 replicates included | 6 replicates included | 6 replicates ND | 6 replicates ND |
| 5.0 | 6 replicates included | 6 replicates included | 6 replicates included | 6 replicates ND | 6 replicates ND |
| 10.0 | 6 replicates included | 6 replicates included | 6 replicates included | 6 replicates ND | 6 replicates ND |
| *Abbreviations: ND, not detected.* | | | | | |

| **Supplementary Table VIII. Extracellular experimental replicates for 4-hour treatment of β-alanine in H9c2 cells.** | | | | | |
| --- | --- | --- | --- | --- | --- |
| **β-alanine concentration (mM)** | **Extracellular taurine** | **Extracellular β-alanine** | **Extracellular L-histidine** | **Extracellular carnosine** | **Extracellular anserine** |
| 0.0 | 6 replicates included | Not analysed | 6 replicates included | 6 replicates ND | 6 replicates ND |
| 0.1 | 6 replicates included | Not analysed | 6 replicates included | 6 replicates ND | 6 replicates ND |
| 0.5 | 6 replicates included | Not analysed | 6 replicates included | 6 replicates ND | 6 replicates ND |
| 1.0 | 6 replicates included | Not analysed | 6 replicates included | 6 replicates ND | 6 replicates ND |
| 5.0 | 6 replicates included | Not analysed | 6 replicates included | 6 replicates ND | 6 replicates ND |
| 10.0 | 6 replicates included | Not analysed | 6 replicates included | 6 replicates ND | 6 replicates ND |
| *Abbreviations: ND, not detected.* | | | | | |

| **Supplementary Table IX. Intracellular experimental replicates for 24-hour treatment of β-alanine in H9c2 cells.** | | | | | |
| --- | --- | --- | --- | --- | --- |
| **β-alanine concentration (mM)** | **Intracellular taurine** | **Intracellular β-alanine** | **Intracellular L-histidine** | **Intracellular carnosine** | **Intercellular anserine** |
| 0.0 | 6 replicates included | 6 replicates included | 2 replicates included  Issue with 4 replicates | 6 replicates ND | 6 replicates ND |
| 0.1 | 6 replicates included | 6 replicates included | 4 replicates included  Issue with 2 replicates | 6 replicates ND | 6 replicates ND |
| 0.5 | 6 replicates included | 6 replicates included | 2 replicates included  Issue with 4 replicates | 6 replicates ND | 6 replicates ND |
| 1.0 | 6 replicates included | 6 replicates included | 5 replicates included  Issue with 1 replicate | 6 replicates ND | 6 replicates ND |
| 5.0 | 4 replicates included  2 replicates ND | 6 replicates included | 3 replicates included  Issue with 3 replicates | 6 replicates ND | 6 replicates ND |
| 10.0 | 6 replicates ND | 6 replicates included | 4 replicates included  Issue with 2 replicates | 6 replicates ND | 6 replicates ND |
| *Abbreviations: ND, not detected.* | | | | | |

| **Supplementary Table X. Extracellular experimental replicates for 24-hour treatment of β-alanine in H9c2 cells.** | | | | | |
| --- | --- | --- | --- | --- | --- |
| **β-alanine concentration (mM)** | **Extracellular taurine** | **Extracellular β-alanine** | **Extracellular L-histidine** | **Extracellular carnosine** | **Extracellular anserine** |
| 0.0 | 5 replicates included  Issue with 1 replicate | Not analysed | 6 replicates included | 6 replicates ND | 6 replicates ND |
| 0.1 | 6 replicates included | Not analysed | 6 replicates included | 6 replicates ND | 6 replicates ND |
| 0.5 | 6 replicates included | Not analysed | 6 replicates included | 6 replicates ND | 6 replicates ND |
| 1.0 | 6 replicates included | Not analysed | 6 replicates included | 6 replicates ND | 6 replicates ND |
| 5.0 | 6 replicates included | Not analysed | 6 replicates included | 6 replicates ND | 6 replicates ND |
| 10.0 | 6 replicates included | Not analysed | 6 replicates included | 6 replicates ND | 6 replicates ND |
| *Abbreviations: ND, not detected.* | | | | | |

| **Supplementary Table XI. Intracellular experimental replicates for 72-hour treatment of β-alanine in H9c2 cells.** | | | | | |
| --- | --- | --- | --- | --- | --- |
| **β-alanine concentration (mM)** | **Intracellular taurine** | **Intracellular β-alanine** | **Intracellular L-histidine** | **Intracellular carnosine** | **Intercellular anserine** |
| 0.0 | 6 replicates included | 6 replicates included | 5 replicates included  1 replicate ND | 6 replicates ND | 6 replicates ND |
| 0.1 | 6 replicates included | 6 replicates included | 6 replicates included | 6 replicates ND | 6 replicates ND |
| 0.5 | 6 replicates included | 6 replicates included | 5 replicates included  1 replicate ND | 6 replicates ND | 6 replicates ND |
| 1.0 | 6 replicates included | 6 replicates included | 6 replicates included | 6 replicates ND | 6 replicates ND |
| 5.0 | 6 replicates included | 6 replicates included | 6 replicates included | 6 replicates ND | 6 replicates ND |
| 10.0 | 4 replicates included  2 replicates ND | 6 replicates included | 4 replicates included  2 replicates ND | 6 replicates ND | 6 replicates ND |
| *Abbreviations: ND, not detected.* | | | | | |

| **Supplementary Table XII. Extracellular experimental replicates for 72-hour treatment of β-alanine in H9c2 cells.** | | | | | |
| --- | --- | --- | --- | --- | --- |
| **β-alanine concentration (mM)** | **Extracellular taurine** | **Extracellular β-alanine** | **Extracellular L-histidine** | **Extracellular carnosine** | **Extracellular anserine** |
| 0.0 | 6 replicates ND | Not analysed | 6 replicates included | 6 replicates ND | 6 replicates ND |
| 0.1 | 3 replicates included  3 replicates ND | Not analysed | 6 replicates included | 6 replicates ND | 6 replicates ND |
| 0.5 | 6 replicates included | Not analysed | 6 replicates included | 6 replicates ND | 6 replicates ND |
| 1.0 | 6 replicates included | Not analysed | 6 replicates included | 6 replicates ND | 6 replicates ND |
| 5.0 | 6 replicates included | Not analysed | 6 replicates included | 6 replicates ND | 6 replicates ND |
| 10.0 | 6 replicates included | Not analysed | 6 replicates included | 6 replicates ND | 6 replicates ND |
| *Abbreviations: ND, not detected.* | | | | | |
