# Supplementary material for: Carnosine‐Related Metabolism in Rat Cardiomyocytes and Human Heart Tissue
Source: FASEB J. 2026 Jul 15;40(14):e72033. doi: 10.1096/fj.202504676R (PMC13371992; doi:10.1096/fj.202504676R)
Supplement: Supplementary file 2 — Figure SI: Ponceau staining loading controls for Western Blot membranes: (A) CARNS1, (B) CNDP2, (C) TAUT, (D) PAT1, (E) PHT1, and (F) PHT2. [file FSB2-40-e72033-s001.pdf]

Supplementary Material 2

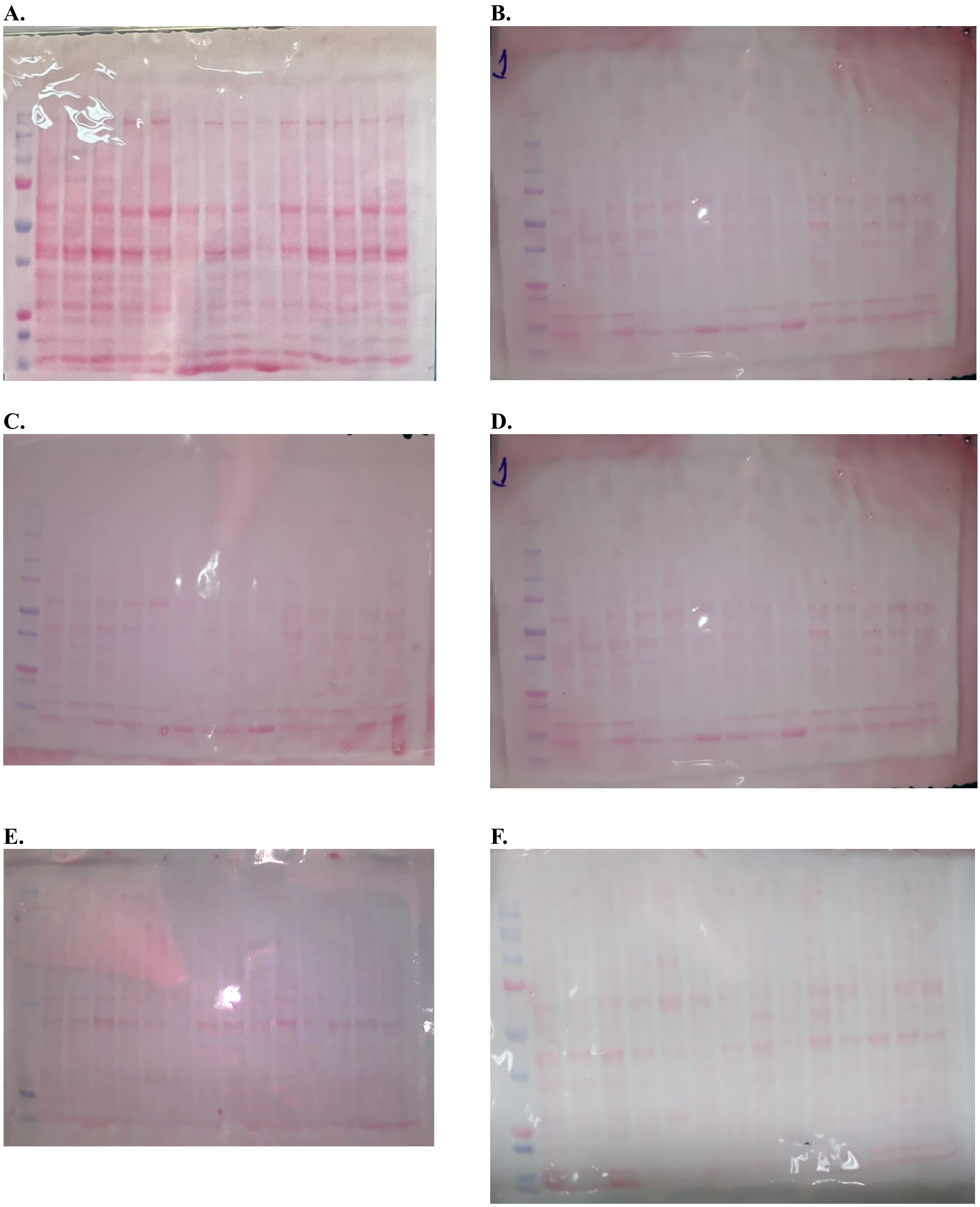

Supplementary Figure I. Ponceau staining loading controls for Western Blot membranes: A) CARNS1, B) CNDP2, C) TAUT, D) PAT1, E) PHT1, and F) PHT2.
